# Supplementary material for: IFN-λ1 Displays Various Levels of Antiviral Activity In Vitro in a Select Panel of RNA Viruses
Source: Viruses. 2021 Aug 12;13(8):1602. doi: 10.3390/v13081602 (PMC8402797; doi:10.3390/v13081602)
Supplement: Supplementary file 1 [file viruses-13-01602-s001.zip › viruses-1302391-supplementary.pdf]

Table S1: Primers and probes used to quantify ISG expression.

Gene	Primer name	Sequence 5'-3'	Product length, bp
Rig-1	qH_RIG1_F1940	GAGCACTTGTGGACGCTTTA	133
	qH_RIG1_R2053	ATACACTTCTGTGCCGGGAG	
	qH_RIG1_O2002	ROX-CCTGGCATATTGACTGGACGTGGC-BHQ2	
OAS-1	qh_OAS1_F	CCAAGGTGGTAAAGGGTGGCT	200
	qh_OAS1_R	CTGGACCTCAAACTTCACGGAAA	
	qh_OAS1_O	Cy3-AGGCCGATCTGACGCTGACCTGGTTGT-BHQ2	
SOCS-1	qh_SOCS-1_F	GGAGCATGCGCGAGAGC	149
	qh_SOCS-1_R	CCTGGTTGTGTGCTACCATCC	
	qh_SOCS-1_O	ROX-CCCTCCGGCTGGCCCCTTCTGTA-BHQ2	
PKR	q_hEIF2AK2_F	GAAAGCGAACAAGGAGTAAGGGA	175
	qh_EIF2AK2_R	CCATCCCGTAGGTCTGTGAAA	
	qh_EIF2AK2_O	Cy5-AGCCCCAAAGCGTAGAGGTCCACTTCC-BHQ3	
MxA	NS02F_hMxA	GAGACAATCGTGAAACAGCAAATCA	105
	NS02R_hMxA	TATCGAAACATCTGTGAAAGCAAGC	
	NS02P.a_hMxA	FAM-CACTGGAAGAGCCGGCTGTGGATATG-BHQ1	

Table S2: Primers and probes used to quantify viral RNA replication.

Virus	Viral gene	Primer name	Sequence 5'-3'	Reference
IVA	*M-protein gene*	InfA Forward	GACCRATCCTGTCACCTCTGAC	[S1]
		InfA Reverse	AGGGCATTYTGGACAAKCGTCTA	
		InfA Probe	FAM-TGCAGTCCTGCGTCACTGGGCACG-BHQ1	
AdV	*hexon*	Adeno-F	GCCACGGTGGGGTTTCTAAACTT	[S2]
		Adeno-R	GCCCCAGTGGTCTTACATGCACATC	
		Adeno-O	FAM-TGCACCAGACCCGGGCTCAGGTACTCCGA-BHQ1	

S1) World Health Organization, 2009. CDC protocol of real-time RT-PCR for swine influenza (H1N1). World Health Organization, Geneva, Switzerland. http://www.who.int/csr/resources/publications/swine-flu/CDCrealtimeRTPCRprotocol_20090428.pdf. Accessed 8 May 2009.
S2) Heim, A., Ebnet, C., Harste, G., & Pring-Åkerblom, P. (2003). Rapid and quantitative detection of human adenovirus DNA by real-time PCR. *Journal of medical virology*, *70*(2), 228-239.

Table S3: Statistical analysis of qPCR data (dCt) reflecting reproduction kinetics of IVA and AdV.

		3 hpi	8 hpi	18 hpi	24 hpi
**IVA**	**P value**	**0,0034**	**0,0055**	**0,0211**	**0,0110**
	**Significantly different?**	Yes (**)	Yes (**)	Yes (*)	Yes (*)
	**Mean ± SEM of w/o treatment**	10,59 ± 0,08145 N=3	0,1000 ± 0,6950 N=3	-1,217 ± 0,3075 N=3	-1,247 ± 0,5948 N=3
	**Mean ± SEM of IFN-l500 ng/ml**	13,02 ± 0,3820 N=3	3,917 ± 0,06888 N=3	0,7133 ± 0,4240 N=3	1,700 ± 0,2821 N=3
	**95% confidence interval**	1,349 to 3,518	1,878 to 5,756	0,4759 to 3,384	1,119 to 4,774
	**R square**	0,9066	0,8819	0,7725	0,8336
**AdV**	**P value**	**0,6224**	**0,5077**	**0,6013**	**0,2114**
	**Significantly different?**	No (ns)	No (ns)	No (ns)	No (ns)
	**Mean ± SEM of w/o treatment**	13,71 ± 0,1010 N=3	14,53 ± 1,079 N=3	1,200 ± 0,3750 N=3	-1,983 ± 0,9644 N=3
	**Mean ± SEM of IFN-l500 ng/ml**	13,98 ± 0,5062 N=3	15,67 ± 1,123 N=3	1,420 ± 0,1007 N=3	-0,5067 ± 0,2390 N=3

	**95% confidence interval**	-1,158 to 1,708	-3,193 to 5,456	-0,8581 to 1,298	-1,282 to 4,235
	**R square**	0,06626	0,1166	0,07429	0,3557

N — sample size; NS — no significant differences; ** — P Value < 0.01; * — P Value < 0.05

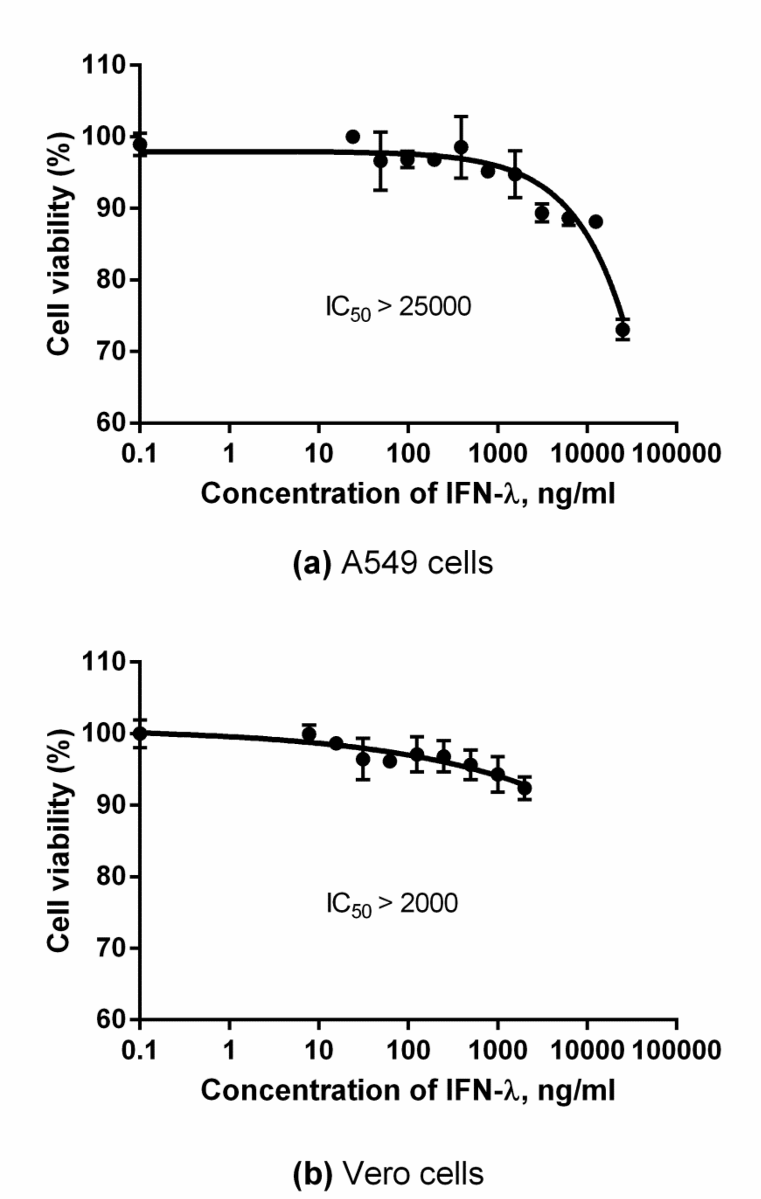

Figure S1. Dose-dependent cytotoxicity of IFN-λ1 in A549 (a) and Vero (b) cells after 24 hrs of exposure (MTT assay). Cells were seeded into 96-well plates and allowed to attach overnight. Afterwards, media were replaced with 100 μl of IFN-λ1 at different concentrations and incubated for 24 hrs. After incubation, 10 μl of MTT (5 mg/ml in PBS) was added, with further incubation for 2 hrs. Supernatants were subsequently removed; cells and MTT crystals were dissolved in DMSO. The amount of blue formazan generated was then determined ($OD_{570\ nm}$). The results are calculated as percent viability in relation to untreated cells. Data are presented as mean ± SD for four biological replicates.

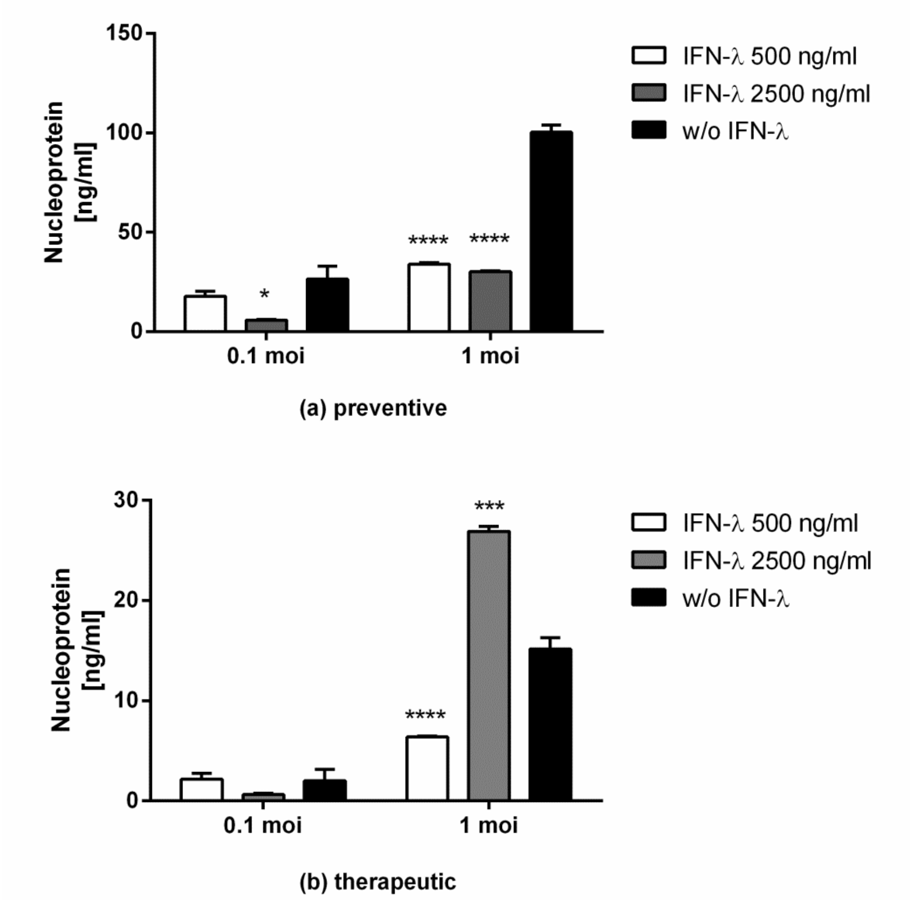

Figure S2: Replication of A/California/07/09 (H1N1pdm09) in A549 cells in response to IFN-λ1 treatment: (a) 'preventive' treatment, (b) 'therapeutic' treatment.

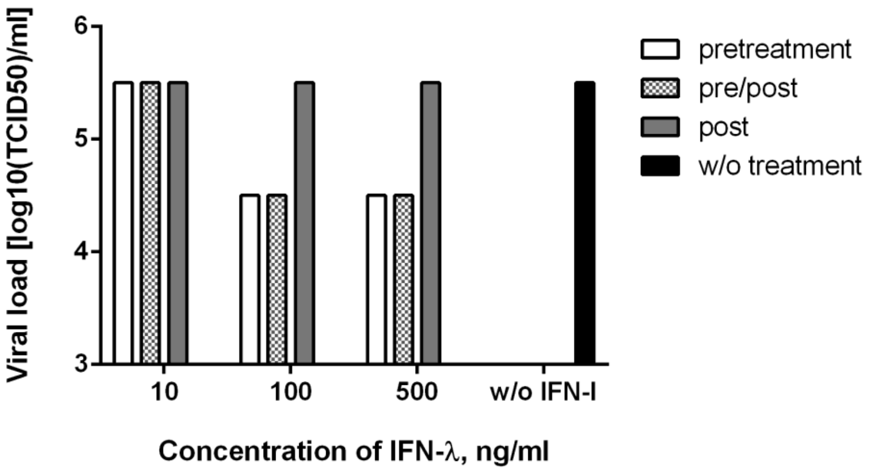

Figure S3: Inhibition of influenza A virus replication. Vero cells were infected with A/PR/8/34 (H1N1) without subsequent addition of trypsin (single-cycle IVA replication). Viral titers were determined at 72 hpi.

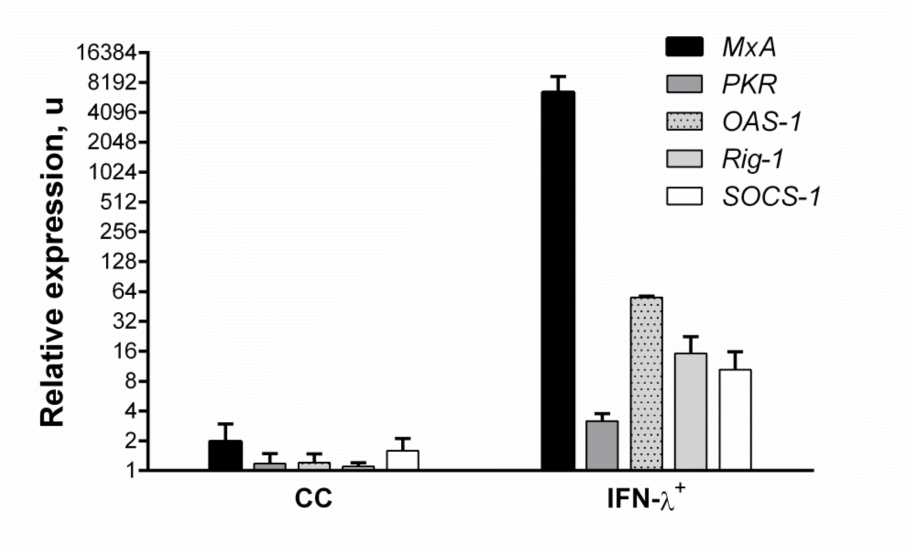

Figure S4: Interferon stimulated gene expression in Vero cells in response to IFN-λ1 stimulation.

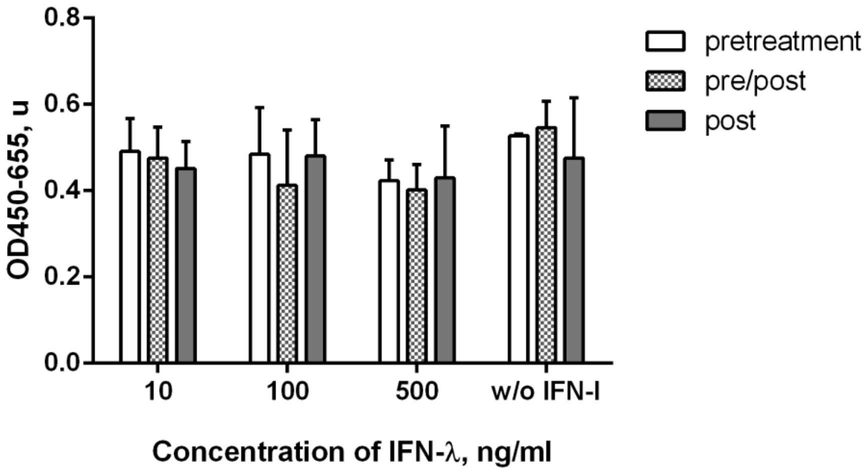

Figure S5: Replication of AdV (serotype 5) in A549 cells in response to IFN-λ1 treatment. Viral antigen was measured at 24 hpi by In-Cell ELISA with anti-hexon antibodies.
